# Supplementary material for: Differential Effects of Overexpression of Wild Type and Kinase-Dead MELK in Fibroblasts and Keratinocytes, Potential Implications for Skin Wound Healing and Cancer
Source: Int J Mol Sci. 2023 Apr 30;24(9):8089. doi: 10.3390/ijms24098089 (PMC10179274; doi:10.3390/ijms24098089)
Supplement: Supplementary file 1 [file ijms-24-08089-s001.zip › ijms-2337760-supplementary.pdf]

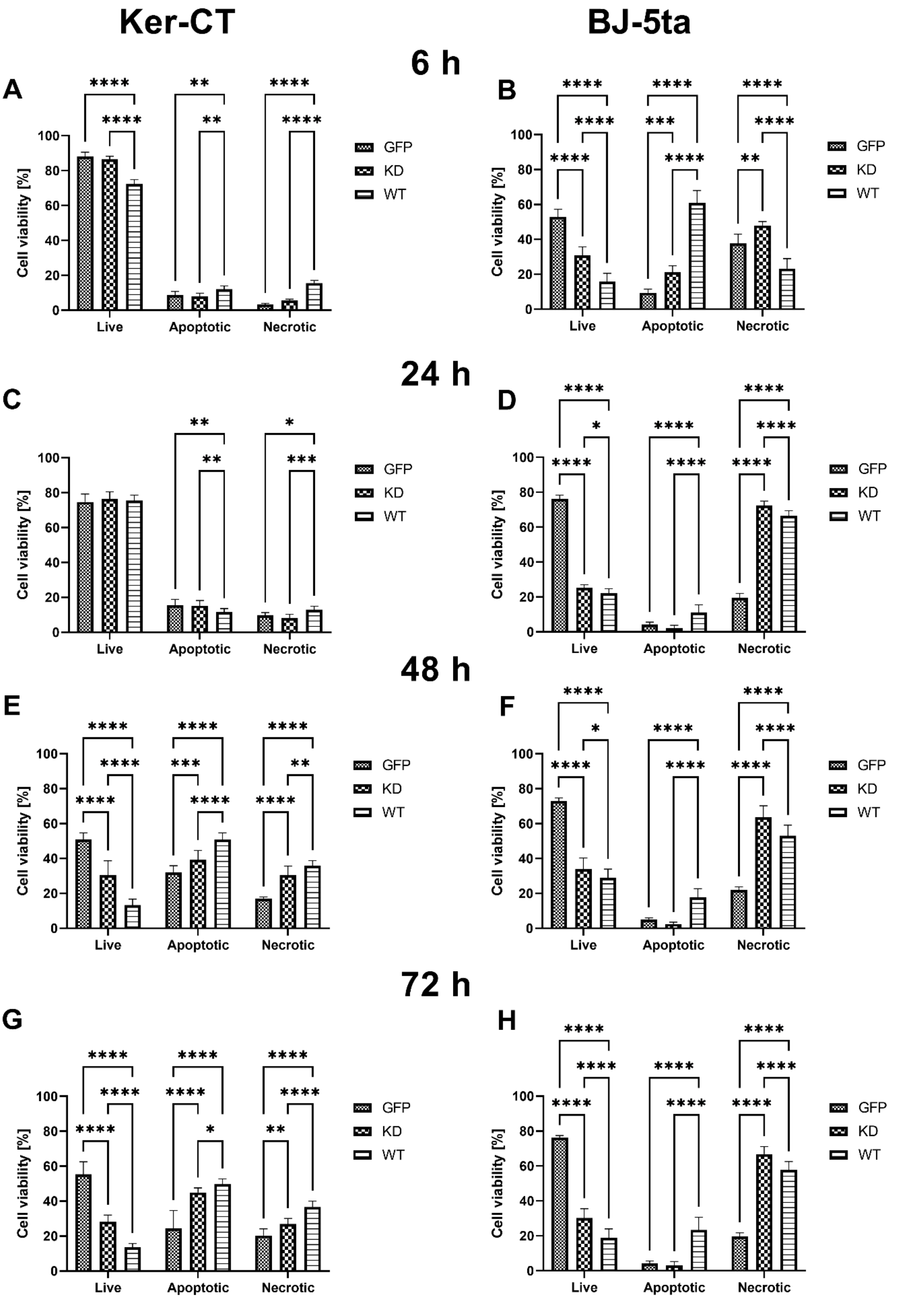

Figure S1. Effects of MELK WT and MELK KD overexpression on cell viability in BJ-5ta fibroblasts and Ker-CT keratinocytes. A – percentage of live, apoptotic and necrotic keratinocytes 6h after transfection

with MELK WT, MELK KD and empty plasmid. B – percentage of live, apoptotic and necrotic fibroblasts 6h after transfection with MELK WT, MELK KD and empty plasmid. C – percentage of live, apoptotic and necrotic keratinocytes 24h after transfection with MELK WT, MELK KD and empty plasmid. D – percentage of live, apoptotic and necrotic fibroblasts 24h after transfection with MELK WT, MELK KD and empty plasmid. E – percentage of live, apoptotic and necrotic keratinocytes 48h after transfection with MELK WT, MELK KD and empty plasmid. F – percentage of live, apoptotic and necrotic fibroblasts 48h after transfection with MELK WT, MELK KD and empty plasmid. G – percentage of live, apoptotic and necrotic keratinocytes 72h after transfection with MELK WT, MELK KD and empty plasmid. H – percentage of live, apoptotic and necrotic fibroblasts 72h after transfection with MELK WT, MELK KD and empty plasmid. Results are presented as the mean SD of at least three independent experiments ($n \geq 9$). * - $p \leq 0.05$, ** - $p \leq 0.01$, *** - $p \leq 0.001$, **** - $p \leq 0.0001$

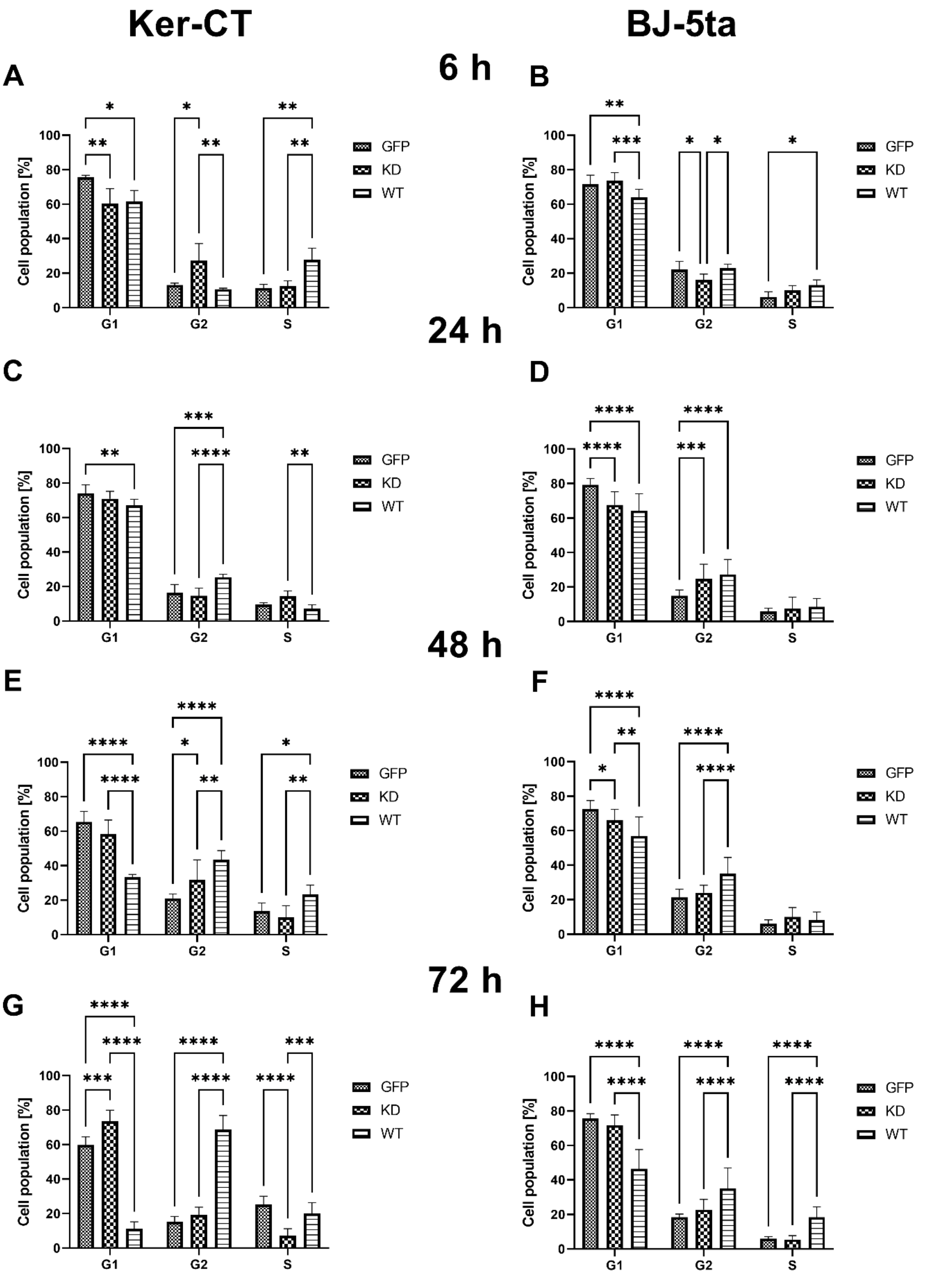

Figure S2. Effects of MELK WT and MELK KD overexpression on cell cycle distribution in BJ-5ta fibroblasts and Ker-CT keratinocytes. A – percentage of keratinocytes in G1, G2 and S phase 6h after

transfection with MELK WT, MELK KD and empty plasmid. B – percentage of fibroblasts in G1, G2 and S phase 6h after transfection with MELK WT, MELK KD and empty plasmid. C – percentage of keratinocytes in G1, G2 and S phase 24h after transfection with MELK WT, MELK KD and empty plasmid. D – percentage of fibroblasts in G1, G2 and S phase 24h after transfection with MELK WT, MELK KD and empty plasmid. E – percentage of keratinocytes in G1, G2 and S phase 48h after transfection with MELK WT, MELK KD and empty plasmid. F – percentage of fibroblasts in G1, G2 and S phase 48h after transfection with MELK WT, MELK KD and empty plasmid. G – percentage of keratinocytes in G1, G2 and S phase 72h after transfection with MELK WT, MELK KD and empty plasmid. H – percentage of fibroblasts in G1, G2 and S phase 72h after transfection with MELK WT, MELK KD and empty plasmid. Results are presented as the mean SD of at least three independent experiments ($n \geq 9$). * - $p \leq 0.05$, ** - $p \leq 0.01$, *** - $p \leq 0.001$, **** - $p \leq 0.0001$

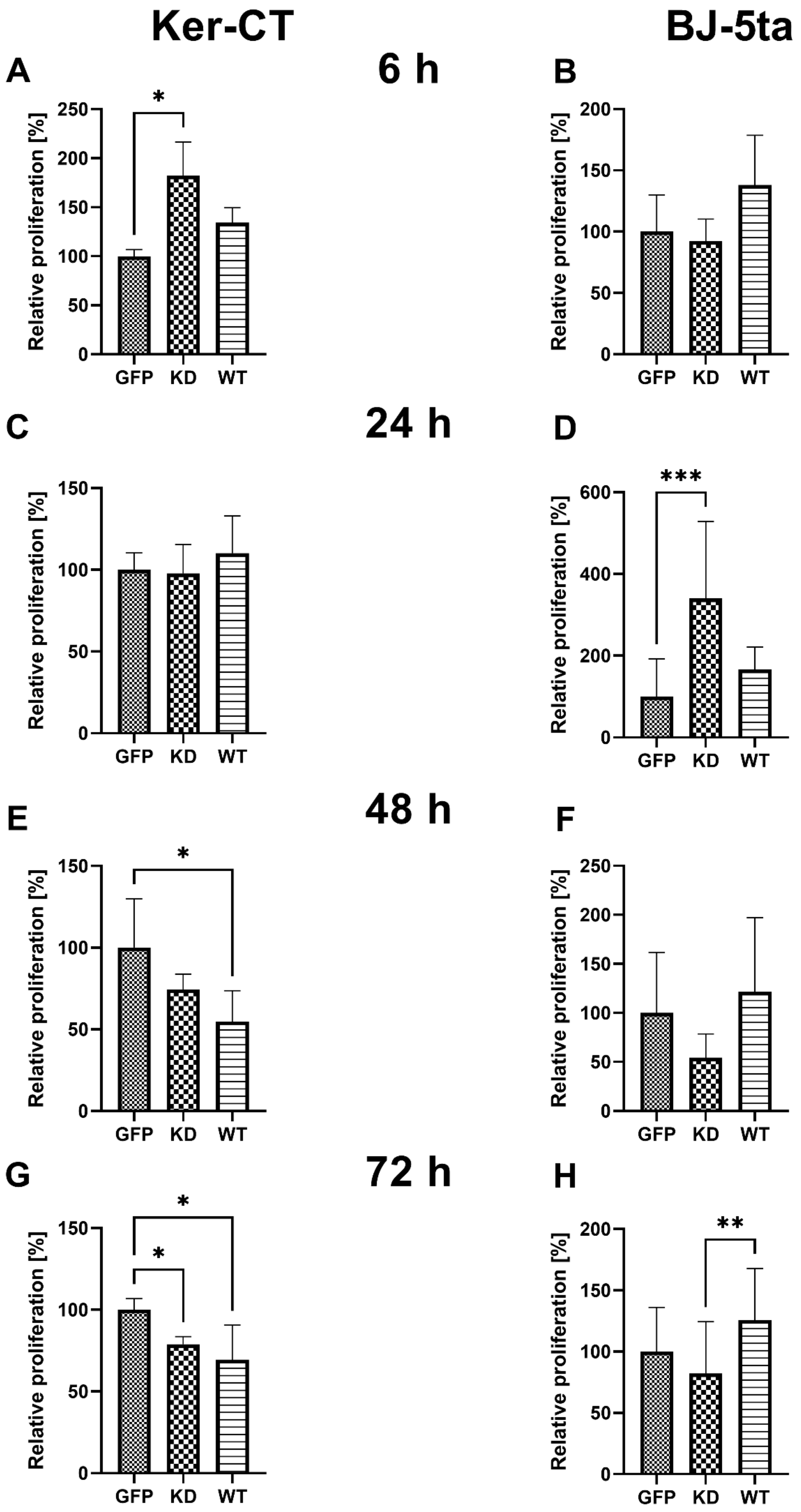

Ker-CT
BJ-5ta
6 h
24 h
48 h
72 h
A
B
C
D
E
F
G
H
Relative proliferation [%]
GFP
KD
WT
*
***
**

Figure S3. Effects of MELK WT and MELK KD overexpression on proliferation rate in BJ-5ta fibroblasts and Ker-CT keratinocytes. A – relative, compared to empty vector transfected cells, proliferation of keratinocytes 6h after transfection with MELK WT and MELK KD plasmids. B – relative, compared to empty vector transfected cells, proliferation of fibroblasts 6h after transfection with MELK WT and MELK KD plasmids. C – relative, compared to empty vector transfected cells, proliferation of keratinocytes 24h after transfection with MELK WT and MELK KD plasmids. D – relative, compared to empty vector transfected cells, proliferation of fibroblasts 24h after transfection with MELK WT and MELK KD plasmids. E – relative, compared to empty vector transfected cells, proliferation of keratinocytes 48h after transfection with MELK WT and MELK KD plasmids. F – relative, compared to empty vector transfected cells, proliferation of fibroblasts 48h after transfection with MELK WT and MELK KD plasmids. G – relative, compared to empty vector transfected cells, proliferation of keratinocytes 72h after transfection with MELK WT and MELK KD plasmids. H – relative, compared to empty vector transfected cells, proliferation of fibroblasts 72h after transfection with MELK WT and MELK KD plasmids. Results are presented as the mean SD of at least three independent experiments ($n \geq 9$). * - $p \leq 0.05$, ** - $p \leq 0.01$, *** - $p \leq 0.001$, **** - $p \leq 0.0001$

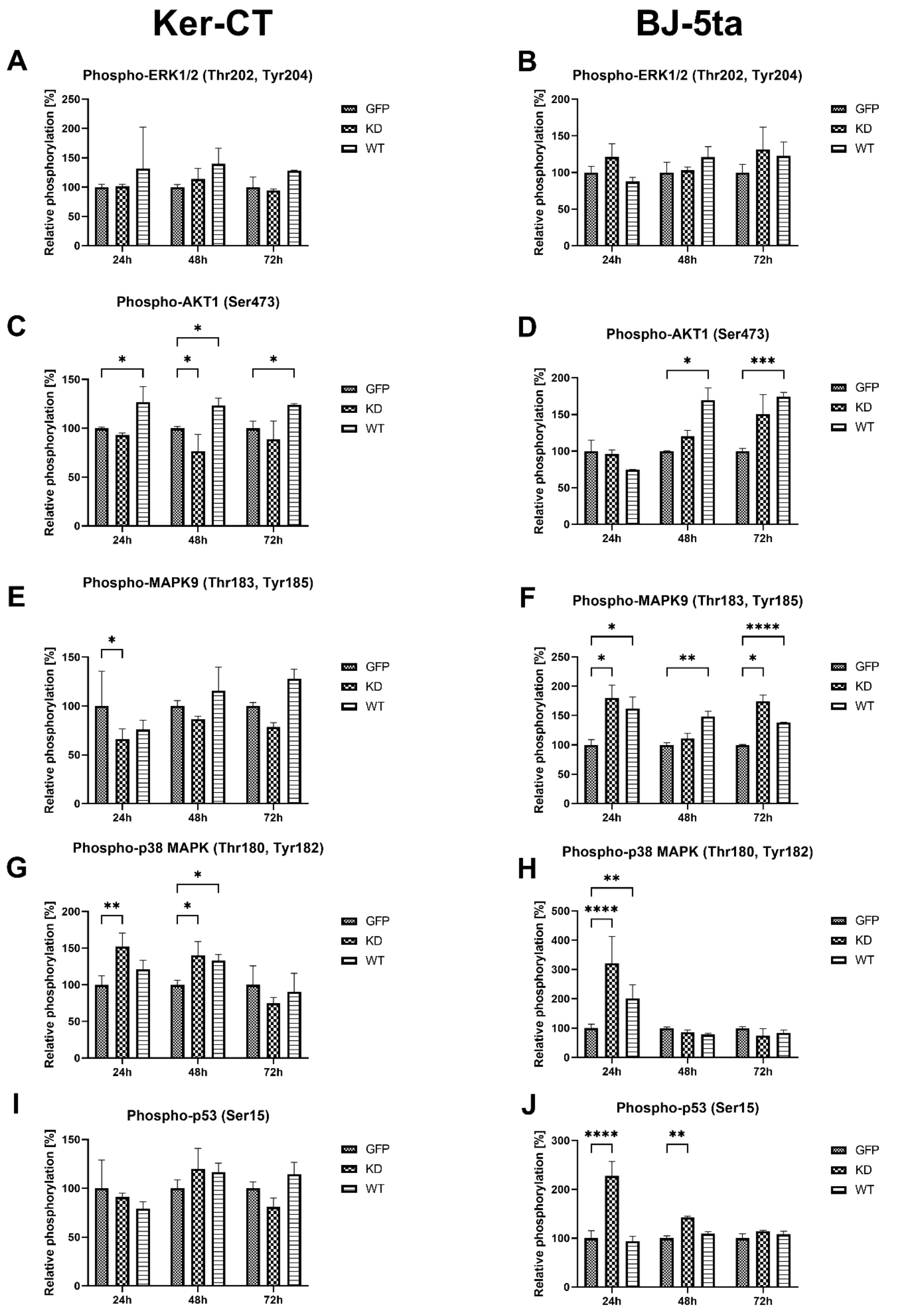

Ker-CT
BJ-5ta
A
Phospho-ERK1/2 (Thr202, Tyr204)
B
Phospho-ERK1/2 (Thr202, Tyr204)
C
Phospho-AKT1 (Ser473)
D
Phospho-AKT1 (Ser473)
E
Phospho-MAPK9 (Thr183, Tyr185)
F
Phospho-MAPK9 (Thr183, Tyr185)
G
Phospho-p38 MAPK (Thr180, Tyr182)
H
Phospho-p38 MAPK (Thr180, Tyr182)
I
Phospho-p53 (Ser15)
J
Phospho-p53 (Ser15)
Relative phosphorylation [%]
24h
48h
72h
GFP
KD
WT

Figure S4. Effects of MELK WT and MELK KD overexpression on phosphorylation of ERK1/2, AKT1, MAPK9 (JNK), p38 MAPK, and p53 in BJ-5ta fibroblasts. A –– relative, compared to empty vector transfected cells, phosphorylation of ERK1/2 (Thr202, Tyr204) in keratinocytes 24h, 48 and 72h after transfection with MELK WT and MELK KD plasmids. B –– relative, compared to empty vector transfected cells, phosphorylation of ERK1/2 (Thr202, Tyr204) in fibroblasts 24h, 48 and 72h after transfection with MELK WT and MELK KD plasmids. C - relative, compared to empty vector transfected cells, phosphorylation of AKT1 (Ser473) in keratinocytes 24h, 48 and 72h after transfection with MELK WT and MELK KD plasmids. D - relative, compared to empty vector transfected cells, phosphorylation of AKT1 (Ser473) in fibroblasts 24h, 48 and 72h after transfection with MELK WT and MELK KD plasmids. E - relative, compared to empty vector transfected cells, phosphorylation of MAPK9/JNK (Thr183, TYR185) in keratinocytes 24h, 48 and 72h after transfection with MELK WT and MELK KD plasmids. F- relative, compared to empty vector transfected cells, phosphorylation of MAPK9/JNK (Thr183, TYR185) in fibroblasts 24h, 48 and 72h after transfection with MELK WT and MELK KD plasmids. G – relative, compared to empty vector transfected cells, phosphorylation of p38 MAPK (Thr180, Tyr182) in keratinocytes 24h, 48 and 72h after transfection with MELK WT and MELK KD plasmids. H– relative, compared to empty vector transfected cells, phosphorylation of p38 MAPK (Thr180, Tyr182) in fibroblasts 24h, 48 and 72h after transfection with MELK WT and MELK KD plasmids. I – relative, compared to empty vector transfected cells, phosphorylation of p53 (Ser15) in keratinocytes 24h, 48 and 72h after transfection with MELK WT and MELK KD plasmids. J – relative, compared to empty vector transfected cells, phosphorylation of p53 (Ser15) in fibroblasts 24h, 48 and 72h after transfection with MELK WT and MELK KD plasmids. Results are presented as the mean SD of at least three independent experiments (n = 6). * - $p \leq 0.05$, ** - $p \leq 0.01$, *** - $p \leq 0.001$, **** - $p \leq 0.0001$

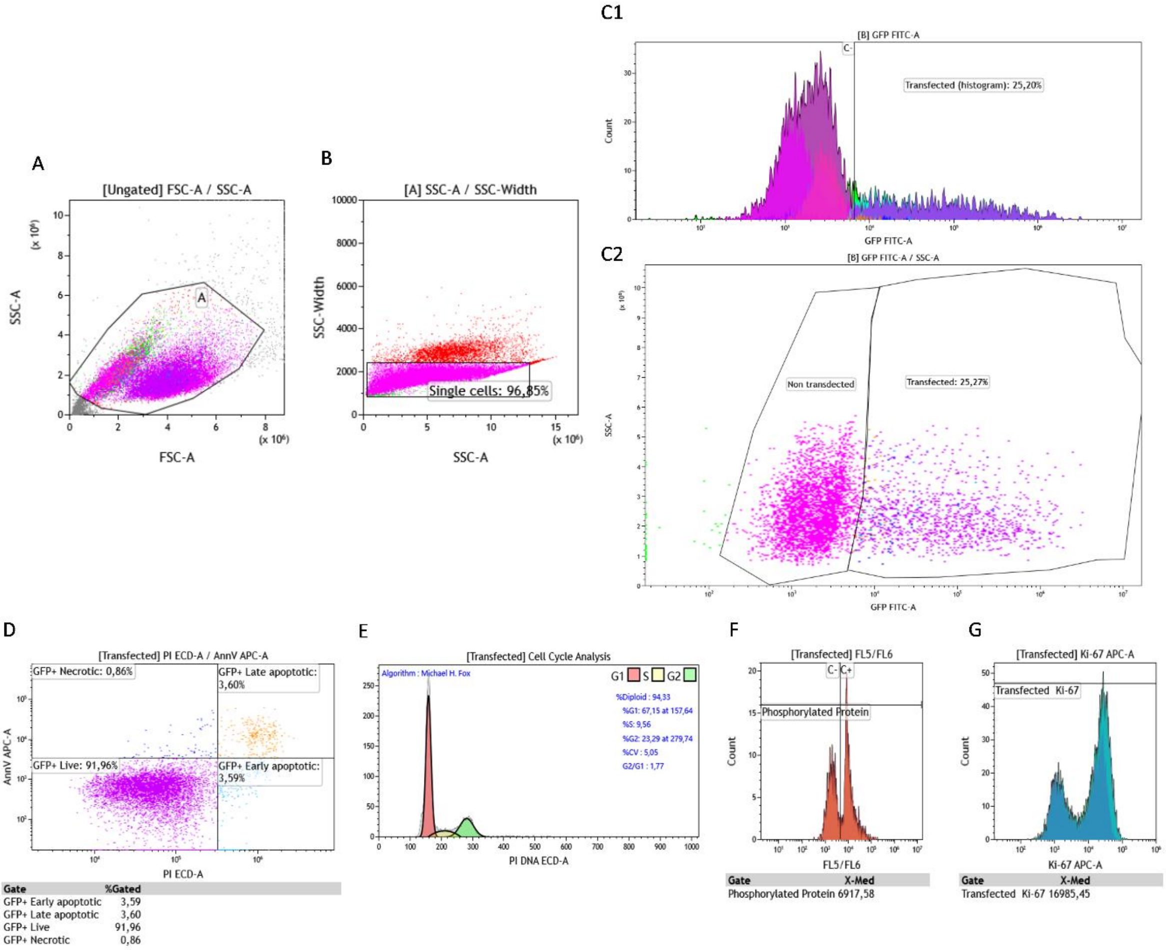

Figure S5. Gating strategy for cytometric experiments using representative plots. A - All cells gating using forward and side scatters. B - Doublet discrimination/single cell selection. C1 and C2 – Transfected cell selection based on the GFP signal. D – Viability evaluation of transfected cells using AnnV and PI staining . E – Cell cycle analysis of transfected cells using FxCycle PI/RNase Staining Solution. F – Cell signaling analysis of transfected cells using phospho-specific antibodies. G – Proliferation analysis of transfected cells using Ki67-APC antibody.
